# Circulating exosomal *miR‐363‐5p* inhibits lymph node metastasis by downregulating *PDGFB* and serves as a potential noninvasive biomarker for breast cancer

**DOI:** 10.1002/1878-0261.13029

**Published:** 2021-06-25

**Authors:** Xin Wang, Tianyi Qian, Siqi Bao, Hengqiang Zhao, Hongyan Chen, Zeyu Xing, Yalun Li, Menglu Zhang, Xiangzhi Meng, Changchang Wang, Jie Wang, Hongxia Gao, Jiaqi Liu, Meng Zhou, Xiang Wang

**Affiliations:** ^1^ Department of Breast Surgical Oncology National Cancer Center/National Clinical Research Center for Cancer/Cancer Hospital Chinese Academy of Medical Sciences and Peking Union Medical College Beijing China; ^2^ Peking Union Medical College Chinese Academy of Medical Sciences Beijing China; ^3^ School of Biomedical Engineering School of Ophthalmology & Optometry and Eye Hospital Wenzhou Medical University China; ^4^ Department of Orthopedic Surgery Peking Union Medical College Hospital Peking Union Medical College and Chinese Academy of Medical Sciences Beijing China; ^5^ Beijing Key Laboratory for Genetic Research of Skeletal Deformity China; ^6^ State Key Laboratory of Molecular Oncology National Cancer Center/National Clinical Research Center for Cancer/Cancer Hospital Chinese Academy of Medical Sciences and Peking Union Medical College Beijing China; ^7^ Department of Breast Surgery The Affiliated Yantai Yuhuangding Hospital of Qingdao University China

**Keywords:** breast cancer, circulating exosome, lymph node metastasis, *miR‐363‐5p*, miRNA, PDGFB

## Abstract

Sentinel lymph node (LN) biopsy is currently the standard procedure for clinical LN‐negative breast cancer (BC) patients but it is prone to false‐negative results and complications. Thus, an accurate noninvasive approach for LN staging is urgently needed in clinical practice. Here, circulating exosomal microRNA (miRNA) expression profiles in peripheral blood from BC patients and age‐matched healthy women were obtained and analyzed. We identified an exosomal miRNA, *miR‐363‐5p*, that was significantly downregulated in exosomes from plasma of BC patients with LN metastasis which exhibited a consistent decreasing trend in tissue samples from multiple independent datasets. Plasma exosomal *miR‐363‐5p* achieved high diagnostic performance in distinguishing LN‐positive patients from LN‐negative patients. The high *miR‐363‐5p expression* level was significantly correlated with improved overall survival. Functional assays demonstrated that exosomal *miR‐363‐5p* modulates platelet‐derived growth factor (PDGF) signaling activity by targeting PDGFB to inhibit cell proliferation and migration. Our study revealed, for the first time, plasma exosomal *miR‐363‐5p* plays a tumor suppressor role in BC and has the potential for noninvasive LN staging and prognosis prediction of BC.

## Graphical abstract

This study identified downregulated circulating exosomal *miR‐363‐5p* in breast cancer (BC) patients with lymph node (LN) metastasis. The *miR‐363‐5p* achieved high performance as a noninvasive predictor of LN metastasis and prognosis. The exosomal *miR‐363‐5p* inhibits metastatic properties and suppresses platelet‐derived growth factor B expression by targeting the 3′‐UTR. Our study provides a potential biomarker for LN staging and prognosis prediction of BC.

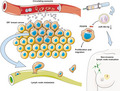

**Abbreviations**
ALN: axillary lymph node
AUC: area under the curve
BC: breast cancer
cDNA: complementary DNA
CHCAMS: Chinese Academy of Medical Sciences
CI: confidence interval
DE: differentially expressed
DMEM: Dulbecco’s modified Eagle’s medium
ER: estrogen receptor
HR: hazard ratio
LN: lymph node
LNM: lymph node metastasis
mRNA: messenger RNA
miRNA: microRNA
NC: negative control
NTA: nanoparticle tracking analysis
PDGF: platelet‐derived growth factor
rRNA: ribosomal RNA
ROC: receiver operating characteristic
SLNB: sentinel lymph node biopsy
TEM: transmission electron microscopy
TPM: total mapped reads

## 1 Introduction

Breast cancer (BC) is the most common malignant tumor in females, with a global annual incidence of 266 120 (30%) and 40 920 (14%) deaths [1]. Axillary lymph node (ALN) metastasis is one of the most important independent risk factors of the prognosis of early BC [2]. Sentinel lymph node (LN) biopsy (SLNB) and ALN dissection are two major procedures for evaluation of ALN status and treatment of ALN metastasis. Currently, SLNB is recommended as the standard approach for ALN evaluation in clinically node‐negative BC patients [3]. Although unnecessary axillary clearance procedures might be spared, sentinel LN could have a false‐negative rate of 7.3%, which leads to patient under‐treatment and causes an increased risk of recurrence [4, 5]. Additionally, the presence of SLNB complications, especially lymphedema, is still inevitable. The incidence rate is 3.5% in BC patients who received SLNB alone without ALN dissection [6]. Thus, it is highly important to develop an accurate and non‐invasive method to identify patients at low risk of ALN metastasis before surgery. Patients without ALN metastasis would benefit vastly if SLNB could be avoided safely using pre‐surgery ALN status evaluation.

Exosomes are 30‐ to 100‐nm microvesicles formed in multivesicular bodies and released into the extracellular environment by most cell types [7]. Abundant studies have shown that exosomes can serve as mediators of cell‐to‐cell communication by delivering cargo molecules, especially nucleic acids, that regulate the tumor microenvironment and promote cancer metastasis and progression [8, 9, 10]. Meanwhile, circulating exosomes of cancer patients were shown to have higher concentrations than in healthy individuals and are considered reliable markers in cancer diagnosis [11, 12]. The circulating exosomes contain a large selection of messenger RNA (mRNA), microRNA (miRNA), long non‐coding RNA, proteins and lipids [13, 14, 15]. The miRNA are small non‐coding RNA that regulate the cellular process by suppressing target mRNA translation and are highly expressed in exosomes [16]. Recent studies have shown that exosomal miRNA exhibit essential biological effects on tumor metastasis [17, 18]. In addition, there is growing evidence suggesting the potential role of exosomal miRNA in the early detection of cancer metastasis [13, 19, 20, 21]. However, the relation between tumor‐derived exosomal miRNA and the LN metastases in BC is still unclear. Biomarkers based on circulating exosomes for clinical applications are not well developed.

We conducted a prior study to investigate the potential use of circulating exosomal miRNA in the detection of LN metastasis (LNM). In this study, small RNA deep sequencing (RNA‐seq) analysis was used, aiming to characterize the miRNA expression landscape in the circulating exosomes from BC patients. The candidate miRNA responsible for BC LNM were generated by comparing the miRNA expression difference between patients with or without LNM. Additionally, candidate miRNA were verified in multiple independent patient datasets. Furthermore, *in* *silico* and experimental studies were performed to identify potentially relevant target genes of the candidate miRNA in order to improve our understanding of mechanisms underlying the LNM in BC.

## 2 Materials and methods

### 2.1 Patient enrollment and sample preparation

All participants were enrolled through the Genetic Investigation of Inherited and Familial Tumor Syndrome study between January 2018 and June 2018 from the Cancer Hospital, Chinese Academy of Medical Sciences (CHCAMS). Patients were eligible for enrollment if they had an evident histologic diagnosis of BC and no distant metastasis. The positive estrogen receptor (ER) was defined as more than 1% of tumor cells staining positive for ER proteins. The HER2‐positive cells were defined as tumor cells that stain strongly (3+) for ERBB2 protein or in which the ERBB2 gene was amplified. Age‐matched healthy women were recruited as a control group. Peripheral blood samples of 10 mL from these BC patients and 10 age‐matched healthy women were collected at CHCAMS. Blood samples were collected in vacuum tubes with EDTA and centrifuged at 3000 ***g*** for 15 min at 4 °C. The collected supernatant (5 mL plasma) was preserved at −80 °C before use. This study was conducted in accordance with the Declaration of Helsinki. All participants signed a written informed consent. Ethics approval for the study was obtained from the Research Ethics Committee of CHCAMS. Two independent BC datasets with miRNA profiles and clinical data were from UCSC Xena Browser (TCGA, http://xena.ucsc.edu/public; *n* = 1044) [22] and Gene Expression Omnibus (GEO, accession number GSE38167, https://www.ncbi.nlm.nih.gov/geo/query/acc.cgi?acc=GSE38167; *n* = 31) [23].

### 2.2 Exosome isolation

The collected plasma was thawed at 37 °C and then centrifuged at 3000 ***g*** for 15 min to remove cell debris. Aspirated supernatant was diluted sevenfold with PBS and centrifuged at 13 000 ***g*** for 30 min [24]. Large particles were removed using 0.22‐μm filters. The collected supernatant was then ultra‐centrifuged at 100 000 ***g***, 4 °C for 2 h (CP100NX; Hitachi, Brea, CA, USA). The pellet containing exosomes was re‐suspended in PBS and ultra‐centrifuged again at 100 000 ***g*** 4 °C for 2 h. The isolated exosomes were re‐suspended in 100 µL PBS after PBS washing for further analysis.

### 2.3 Exosome characterization

The nanoparticle tracking analysis (NTA), transmission electron microscopy (TEM) and western blot analysis using rabbit polyclonal antibody CD63, TSG101 and calnexin were conducted following the previously reported protocols [25].

### 2.4 Exosomal RNA isolation and RNA analyses

The RNA were extracted from plasma‐isolated exosomes using the miRNeasy® Mini kit (Qiagen, cat. No. 217004, Shanghai, China). RNA yields, as well as DNA contamination, were monitored on a 1.50% agarose gel. The NanoDrop 2000 spectrophotometer (ThermoFisher Scientific, Wilmington, DE, USA) was used to assess RNA concentration and purity. The integrity and distribution of RNA were analyzed using the Agilent Bioanalyzer 2100 system with RNA Nano 6000 Assay Kit (Agilent Technologies, Palo Alto, CA, USA).

### 2.5 Library preparation and sequencing

A total amount of 5 ng RNA per sample was depleted of ribosomal RNA (rRNA) using the RiboZero magnetic kit (Epicentre, Madison, WI, USA). Sequencing libraries were then generated using the Ovation® RNA‐Seq System (NuGEN, San Carlos, CA, USA). A total amount of 2.5 μg RNA per sample was used as input material for sample preparation of small RNA libraries. The libraries were generated using the NEB Next Multiplex Small RNA Library Prep Set for Illumina (NEB, Ipswich, MA, USA). The index codes were added to attribute sequences to each sample. Finally, the PCR products were purified using the Agencourt AMPure XP system (Beckman Coulter, Brea, CA, USA). The library quality was evaluated on an Agilent Bioanalyzer 2100 (Agilent Technologies) and quantitative PCR. The cluster of the index‐coded samples was generated by the acBot Cluster Generation System using TruSeq PE Cluster Kitv3‐cBot‐HS (Illumina, San Diego, CA, USA). At last, the sequencing was performed on the Illumina HiSeq platform using the library preparations and paired‐end reads were generated.

### 2.6 Quantitative differential expression analysis of miRNA

The sequence alignment was performed using the Bowtie tool [26] with several databases, including the Silva database (https://www.arb‐silva.de/), the GtRNAdb database (http://gtrnadb.ucsc.edu/), the Rfam database (http://rfam.sanger.ac.uk/) and the Repbase database (http://www.girinst.org/) [27]. Subsequently, the rRNA, transfer RNA, small nuclear RNA, small nucleolar RNA, and other non‐coding RNA were filtered. The miRNA, including known miRNA and novel miRNA, were detected using the remaining reads, in which the novel miRNA were predicted according to the miRbase database and Human Genome (GRCh38), respectively. Read counts of the miRNA were generated from the mapping results and have been standardized as the total mapped reads (TPM) per million. Circulating exosomal miRNA profiles of samples with two conditions were compared using the two‐tailed Student’s *t*‐test, and each miRNA with a log_2_|fold change|> 0.58 and *P* < 0.05 was considered a differential expression. Hierarchical clustering was performed with r package ‘pheatmap’ using the ward.D2 method using R statistical software, version 3.5.1 (R Foundation for Statistical Computing, Vienna, Austria).

### 2.7 Cell culture and transfection

The MCF‐7 cell line was cultured at 37 °C with 5% CO_2_. Dulbecco’s modified Eagle’s medium (DMEM, SH30022.01; HyClone, South Logan, UT, USA) with 10% FBS (FND500, ExCell Bio., Shanghai, China) was applied as a culture medium. In addition, 100 units per milliliter penicillin and 100 μg·mL^−1^ streptomycin (SV30010, HyClone, Logan, UT, USA) were added to DMEM. Until the density reached approximately 50–70%, cells were transfected for 48 h with *miR‐363‐5p* mimic, mock negative control (NC), *miR‐363‐5p* inhibitor or inhibitor NC (Ribo, Guangzhou, China) using Lipofectamine 2000 (Invitrogen, Carlsbad, CA, USA).

### 2.8 RNA extraction and quantification

The miRNA was extracted from MCF‐7 cells with the miRcute miRNA isolation kit (DP501, Tiangen, Beijing, China). Total RNA was extracted from transfected MCF‐7 cells with the total RNA rapid extraction kit (220010, Feijie Biological, Shanghai, China). After quality control, the FastQuant RT kit (KR106, Tiangen, Beijing, China) was used to reverse transcribe the miRNA or RNA sample into complementary DNA (cDNA). qRT‐PCR was performed in an ABI 7300 real‐time PCR system (Applied Biosystems, Foster City, CA, USA). SuperReal PreMix Plus (SYBR Green) mixture (FP205; Tiangen) was applied for reactions. The relative amounts of *miR‐363‐5p* to control *U6* and platelet‐derived growth factor *B* (*PDGFB*) to control GAPDH transcripts were analyzed by the 2^−ΔΔCt^ method. Primers applied were listed as follows: *miR‐363‐5p*: forward: 5′‐CGGGTGGATCACGATG‐3′; reverse: 5′‐CAGTGCAGGGTCCGAGGTAT‐3′; U6: forward: 5′‐CTCGCTTCGGCAGCACA‐3′; reverse: 5′‐AACGCTTCACGAATTTGCGT‐3′ [28].

### 2.9 Cell proliferation assay

The MCF‐7 cells were planted in 96‐well plates with a density of 5 × 10^3^ cells per well. The proliferation of cells at 0, 24, 48 and 72 h after transfection was examined using the CCK‐8 proliferation assay kit (MA2018‐L, Meilunbio, Dalian, China). At every time phase, 10 μL of CCK‐8 reagent was added to the medium. Absorbance at 450 nm was measured after 3 h of incubation using a microplate spectrometer reader (Molecular Devices, San Jose, CA, USA).

### 2.10 Transwell migration assay and colony formation assay

After transfection with *miR‐363‐5p* or NC for 48 h, MCF‐7 cell was washed twice with FBS‐free medium, and then re‐suspended in FBS‐free medium at a density of 1 × 10^5^ cells·mL^−1^. Transwell chamber (pore size 8.0 μm, 3422; Corning Costar, Cambridge, MA, USA) pretreated with the FBS‐free medium was placed in a 24‐well plate. After removing the pretreatment medium, 600 μL 10% FBS‐containing medium was added to the lower chamber and a 100‐μL cell suspension to the upper chamber was added with. After incubating for 48 h, the chambers were fixed and stained with methanol and 0.2% crystal violet. After staining, cells on the chamber surface were removed carefully with water and cotton swabs. The number of perforated cells in the outer layer of the basement membrane of each chamber (migrating cells) was counted in five random high‐power fields with a phase‐contrast microscope (NIB‐100F, Nanjing Jiangnan Novel Optics, Nanjing, China).

Cell proliferation capacity was evaluated with colony formation assay using the protocol previously described by Liu *et al*. [29]. After transfection with *miR‐363‐5p* or NC for 48 h, MCF‐7 cells were seeded in 24‐well microplates with approximately 2000 cells per well. After adherent growth of 48 h, the cells were stained with crystal violet solution after methanol fixation and counted using imagej software (NIH, Bethesda, MA, USA). Three parallel experiments were conducted. The results were normalized using the proliferation data to minimize confounding.

### 2.11 Statistical analysis

Analyses were performed with r Statistical Software (version 3.5.3). Pre‐set *P* < 0.05 was defined as statistically significant. Quantitative data were measured as mean ± standard deviation. The comparison of mean values between the two groups was analyzed using Student’s *t*‐test and Mann–Whitney *U*‐test. Pearson’s test was used to evaluate the exosome‐tissue miRNA correlation and miRNA‐target mRNA correlation. Receiver operating characteristic (ROC) curve analysis was used to determine the diagnostic performance, and the area under the curve (AUC) was calculated with the r package ‘ROCit’ [30]. The Kaplan–Meier method and log‐rank test were applied to compare survival differences and the hazard ratio (HR) and 95% confidence interval (CI) were calculated using the r package ‘Survival’.

## 3 Results

### 3.1 Characterization of exosomes from the plasma of breast cancer patients

In this study, 10 BC (Luminal‐like) patients and 10 age‐matched healthy women were enrolled. Clinical information about the patients is listed in Table 1. Ten BC patients were further divided into two groups according to their LN status, namely, four patients with LNM and six patients without LNM. Blood samples were collected from both BC patients and healthy controls. The integrity of exosome preparation was confirmed with TEM followed by western blot. The exosomes isolated from the plasma exhibited the classic cup‐shaped morphology under TEM (Fig. S1A). Exosome markers *TSG101* and *CD63* expression were detected from the exosome isolated from the plasma (Fig. S1B). The NTA indicated that the average size of the vesicles was 105.7 nm and the main peak of particle diameter was at 85.5 nm (Fig. S1C). The results mentioned above demonstrated that the extracellular vesicles isolated from plasma samples are purified exosomes.

**Table 1:** Clinical information of BC patients used in the exosomal cohort. All patients were pathologically diagnosed with ER^+^HER2^−^ stage I–II IDC or DCIS, according to the BC biologic subtype and TNM anatomic stage classification from AJCC UICC (8th edition). AJCC, The American Joint Committee on Cancer; IDC, invasive ductal carcinoma; DCIS, ductal carcinoma *in situ*; M, metastasis; N, lymph node; T, tumor; UICC, Union for International Cancer Control.

Patient	Gender	Age at diagnosis	Histology	Subtype	T stage	N stage	M stage	Stage	LN
A07‐05	F	63	IDC	ER^+^HER2^−^	1c	1a	0	IIA	1/22
A07‐07	F	49	IDC	ER^+^HER2^−^	1b	2a	0	IIA	2/24
A07‐08	F	53	IDC	ER^+^HER2^−^	1c	0(sn)	0	IA	0/6
A07‐09	F	67	IDC	ER^+^HER2^−^	2	0(sn)	0	IIA	0/5
A07‐10	F	38	IDC	ER^+^HER2^−^	2	0(sn)	0	IIA	0/5
A07‐11	F	55	DCIS	ER^+^HER2^−^	is	0(sn)	0	0	0/4
A07‐12	F	57	IDC	ER^+^HER2^−^	1b	1a	0	IIA	1/23
A07‐14	F	59	IDC	ER^+^HER2^−^	1c	0(sn)	0	IA	0/5
A07‐17	F	64	IDC	ER^+^HER2^−^	2	0(sn)	0	IIA	0/6
A07‐18	F	44	IDC	ER^+^HER2^−^	1c	1a	0	IIA	1/24

### 3.2 RNA‐seq identified dysregulated exo‐miRNA in breast cancer patients

**Fig. 1:**
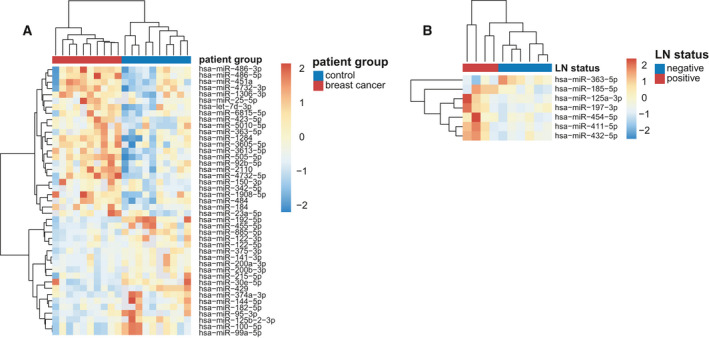
Expression pattern of miRNA in plasma exosomes from BC patients. Heatmap depicting unsupervised hierarchical clustering based on the RNA sequencing expression values of DE plasma exosomal miRNA among BC patients and healthy individuals (A) and BC patients with positive LN and negative LN (B). DE miRNA were filtered by Student’s *t*‐test (*P* < 0.05) with fold change > 1.5 | < 0.67.

To identify exo‐miRNA that play a pivotal role in inducing BC LNM, circulating exosomal miRNA was isolated and profiled using small RNA deep sequencing analysis. A total of 1631 miRNA were mapped in exosomes isolated from plasma samples. To minimize noise and improve accuracy, the miRNA with TPM values of less than five were removed, leaving 367 miRNA for further analysis. Through differential expression analysis, 43 significantly differentially expressed (DE) miRNA were identified in breast tumor exosomes and seven significantly DE miRNA in breast tumor exosomes with LN‐positive status. Figure 1A shows a different expression pattern of the 43 DE miRNA between BC patients and healthy controls. Figure 1B shows a different expression pattern of the seven DE miRNA between BC patients with and without LNM.

### 3.3 Identification of circulating exosomal *miR‐363‐5p* as a potential biomarker of axillary lymph node metastasis and prognosis

**Fig. 2:**
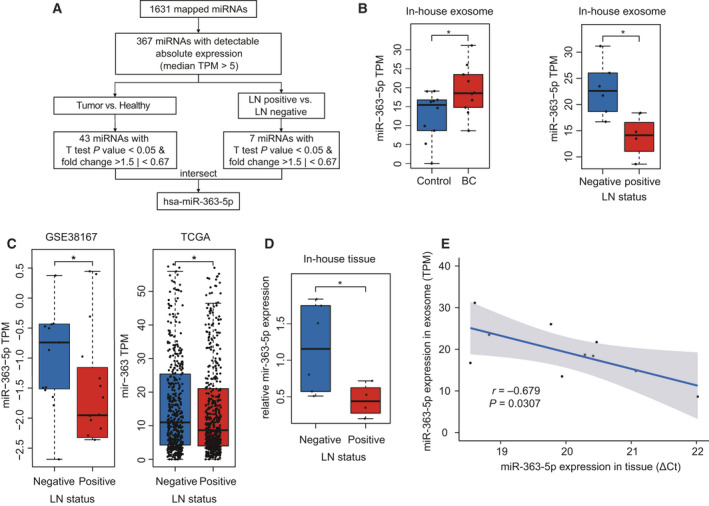
The*miR‐363‐5p* is down‐regulated in circulating exosome and tumor tissue of BC patients with positive LN. (A) Workflow of the filtration procedures used in identifying the potential markers. (B) Exosomal *miR‐363‐5p* expression pattern of in‐house circulating exosome dataset, *P < 0.05 by unpaired Student’s *t*‐test. (C) The presence of LNM at diagnosis is associated with lower expression of *miR‐363‐5p* in BC tissues in public datasets. *P < 0.05 determined by the Mann–Whitney *U‐test*. (D) In‐house expression levels of *miR‐363‐5p* in BC tissues were determined using qRT‐PCR, *P < 0.05 by unpaired Student’s *t*‐test. (E) The *miR‐363‐5p* in circulating exosome displayed consistent expression in matched tumor tissues. Pearson’s correlation analysis was applied.

Integrative profiles analysis indicated that the aberrant expression of exosomal *miR‐363‐5p* is significantly associated with both BC (*P* = 0.047, Mann–Whitney *U*‐test) and ALN metastasis (*P* = 0.019, Mann–Whitney *U*‐test; Fig. 2A,B). Exosomal *miR‐363‐5p* expression was significantly higher in BC patients compared with healthy controls and was significantly lower in LN‐positive patients compared with LN‐negative patients (Fig. 2B). Since the miRNA concentration in exosomes is distinctively related to its cellular abundance [31], we also hypothesized that reliable circulating markers should coordinate with their expression alterations in tumor tissues. To verify the reliability of *miR‐363‐5p*, the expression levels of *miR‐363‐5p* in tumor tissues with and without LN were compared in two external independent patient datasets. The exosomal *miR‐363‐5p* exhibited a consistent expression trend in tissue samples, as observed in plasma samples (Fig. 2C). As shown in Fig. 2C, the expression level of *miR‐363‐5p* is significantly lower in LN‐positive patients than those without LN both in TCGA (*P* = 0.014, Mann–Whitney *U*‐test) and GSE38167 (*P* = 0.013, Mann–Whitney *U*‐test) datasets. Further association analysis showed that a significant expression difference of miR‐363 exists exclusively in ER^+^ BC (Fig. S2), which is consistent with the subtype of in‐house samples. We subsequently profiled the matched expression levels of the miRNA in tumor tissue of 10 BC patients using qRT‐PCR. *MiR‐363‐5p* expression in LN‐negative BC tissue samples was significantly higher compared with LN‐positive patients (*P* = 0.019, Mann–Whitney *U*‐test; Fig. 2D). In addition, our in‐house data showed the exosomal concentrations of *miR‐363‐5p* correlated with its expression in tumor tissue (Pearson’s *r* = −0.679 and *P* = 0.0307, Fig. 2E). Additionally, *miR‐363‐5p* expression in BC tissue was significantly higher than matched para‐tumor tissue (Fig. S3), which also consists with circulating exosomal expressions. These validation analyses indicated that *miR‐363‐5p* is a potential and stable noninvasive biomarker for further investigation.

### 3.4 Performance evaluation and validation of *miR‐363‐5p* in the in‐house and multiple independent datasets

**Fig. 3:**
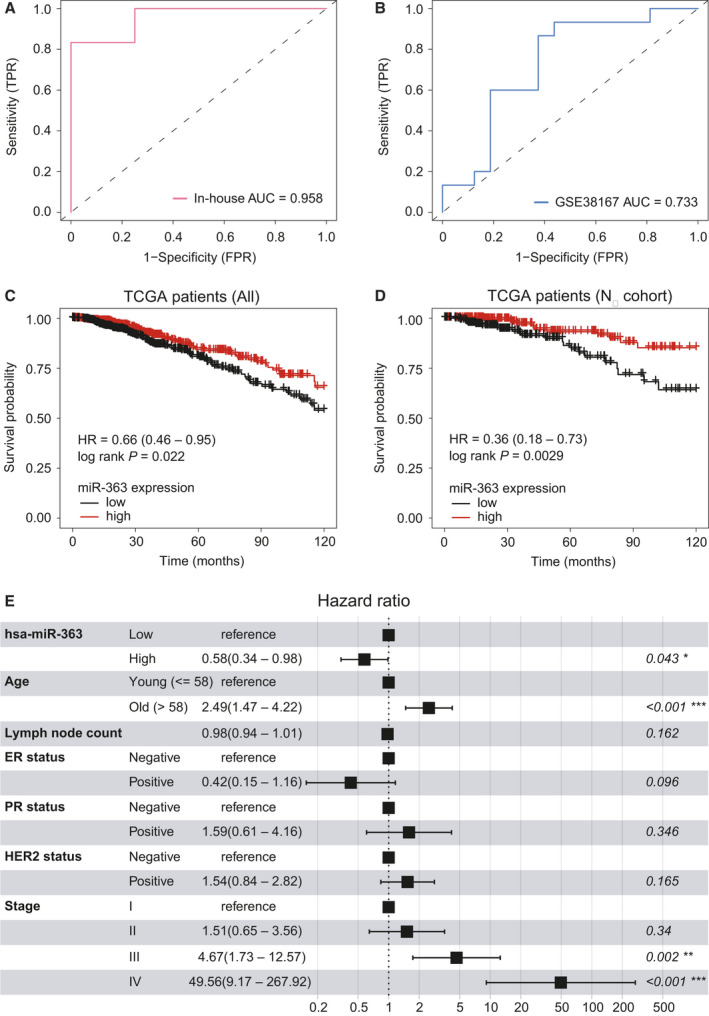
Performance evaluation of *miR‐363‐5p* as a noninvasive predictor of LNM and prognosis. (A,B) The ROC curve of the *miR‐363‐5p* for BC LNM using in‐house circulating exosomal miRNA data and public tissue expression data (GSE38167). (C,D) Kaplan–Meier survival analysis for all TCGA BC patients (C) and patients with negative LN upon first diagnosis (D). Statistical significance was determined by the log‐rank test. (E) Multivariate proportional‐hazards model showed survival impact of *miR‐363‐5p* along with clinical characteristics for TCGA BC patients. The result showed 95% CI of risk of mortality. ***P < 0.001, **P < 0.01 and *P < 0.05 determined by Cox proportional hazards model.

To evaluate retrospectively the predictive power of exosomal *miR‐363‐5p* to detect LNM, we performed ROC analysis and found that the *miR‐363‐5p* achieved high diagnostic performance with an AUC of 0.958 for the in‐house dataset and 0.733 for the GSE38167 dataset, respectively (Fig. 3A,B). These results indicated that low *miR‐363‐5p* expression levels might serve as a potential biomarker for noninvasive LN staging of BC LNM. Furthermore, we assessed the association between *miR‐363‐5p* expression level and survival of BC patients and found that patients with low expression of *miR‐363* had significantly worse overall survival (HR = 0.63, 95% CI 0.45–0.89; *P* = 0.0075, log‐rank test; Fig. 3C). Moreover, in patients with negative LN upon the first diagnosis, low expression of *miR‐363* in primary tumors correlated with a significantly worse outcome (HR = 0.23, 95% CI 0.09–0.60; *P* = 0.00094, log‐rank test; Fig. 3D). Multivariate survival analysis using the proportional hazards model indicated that a high expression level of *miR‐363* could serve as a protective prognostic marker of BC survival (HR = 0.58, *P* = 0.043, Fig. 3E).

### 3.5 *miR‐363‐5p* inhibits metastatic properties of breast cancer cell

**Fig. 4:**
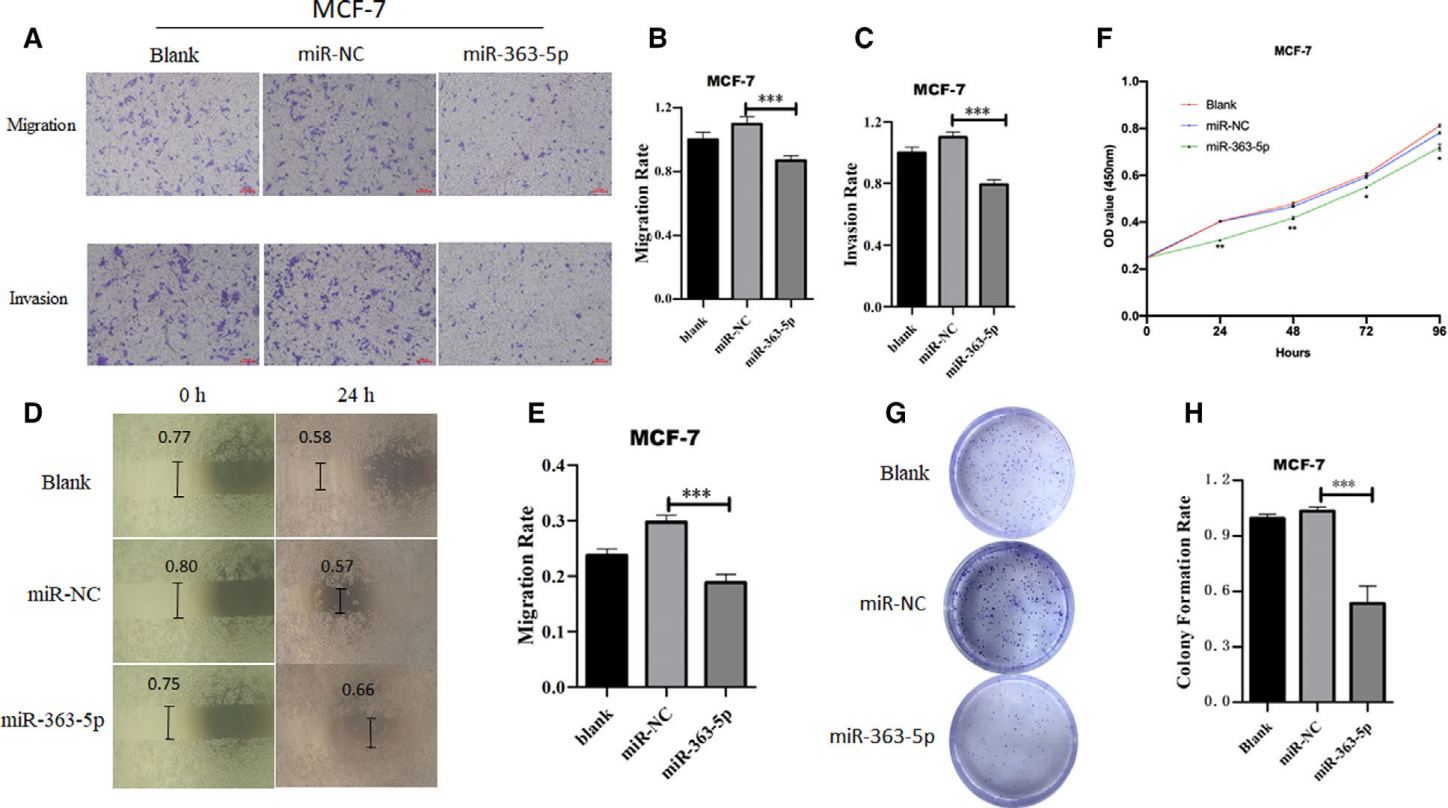
The*miR‐363‐5p* inhibits metastatic properties of the BC cell. (A–C) Transwell migration assay and invasion assay of MCF‐7 cells transfected with *miR‐363‐5p*‐mimic or normal control (NC). Migrated cells were counted using imagej and representative images are shown. (D,E) Wound healing assay of MCF‐7 transfected with *miR‐363‐5p*‐mimic or NC. (F) Proliferation abilities of MCF‐7 transfected with *miR‐363‐5p*‐mimic or NC were detected via CCK‐8 assay. (G,H) Colony formation assay of MCF‐7 transfected with *miR‐363‐5p*‐mimic or NC. Experiments were performed in triplicate and repeated three times with similar results. Scale bar: 20 μm. Results are shown as mean ± SE. ***P < 0.001, **P < 0.01 and *P < 0.05 determined by Student’s *t*‐test.

The *miR‐363‐3p* and *miR‐363‐5p* (miR‐363*) are both mature forms of *miR‐363*. Previous studies have focused on the biological function and pathophysiological significance of *miR‐363‐3p* but few have explored the role of *miR‐363‐5p*, possibly because of its relatively low abundance compared with *miR‐363‐3p*. To investigate the role of circulating exosomal *miR‐363‐5p* in BC progression, we hypothesized that *miR‐363‐5p* influences BC cell mobility. To keep the consistency of the sample subtype, we selected an ER‐positive BC cell line MCF‐7 and transfected BC cells with plasmids overexpressing *miR‐363‐5p* or NC. The results indicated that the overexpression of *miR‐363‐5p* significantly suppresses the migration (Fig. 4A,B), invasion (Fig. 4C–E), proliferation (Fig. 4F) and colony formation (Fig. 4G,H) of MCF‐7 cells.

### 3.6 Exosomal *miR‐363‐5p* modulates platelet‐derived growth factor signaling activity by targeting *PDGFB*

**Fig. 5:**
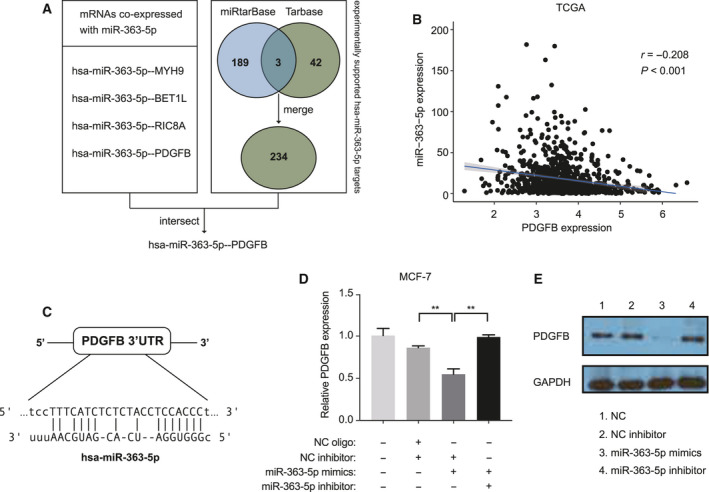
The *miR‐363‐5p* suppresses *PDGFB* expression by binding to its 3′‐UTR. (A) The strategy is applied in target identification. (B) A negative correlation between *miR‐363‐5p* expression and *PDGFB* mRNA levels in BC tissues was analyzed using Pearson’s correlation analysis. (C) The *miR‐363‐5p* binding sequences in *PDGFB* 3′‐UTR. (D) qRT‐PCR. (E) Western blot assay of *PDGFB* expression level of MCF‐7 transfected with *miR‐363‐5p*‐mimic, *miR‐363‐5p*‐inhibitor or normal control. Experiments were performed in triplicate and repeated three times with similar results. Results are shown as mean ± SE. **P < 0.01 determined by Student’s *t*‐test.

To identify reliable targets of *miR‐363‐5p*, we utilized both experimentally validated miRNA‐target interaction databases and co‐expression analysis (Fig. 5A). We analyzed miRNA and mRNA expression profiles of the TCGA BC dataset which yielded four mRNA co‐expressed with *miR‐363‐5p* based on the negative regulation of target gene expression and miRNA level. We also retrieved gene lists of experimentally established targets of *miR‐363‐5p* from two databases (mirTarbase and Tarbase). We merged the results from two databases, producing a list of 234 target genes. Among them, the *PDGFB* oncogene was the only one exhibiting a significant negative correlation (Pearson’s *r* = −0.208, *P* < 0.001) with the *miR‐363‐5p* level in BC tissues from TCGA (Fig. 5B). The target location (Fig. 5C) was produced in the previous study and validated using PAR‐CLIP [32]. These implied that the *PDGFB* oncogene might be a potential functional target of *miR‐363‐5p*. We, therefore, performed qRT‐PCR for validation. Consistent with the bioinformatics analysis, qRT‐PCR and western blot analysis also showed that the expression levels of *PDGFB* mRNA and protein were significantly downregulated by *miR‐363‐5p* overexpression, which is subsequently rescued by *miR‐363‐5p* knockdown as well (Fig. 5D,E). These findings indicated that *miR‐363‐5p* regulates *PDGFB* oncogene expression in BC. The *miR‐363‐5p* deficiency promoted metastasis via facilitating *PDGFB* expression, leading to the overactivity of PDGF signaling in cancer cells.

## 4 Discussion

Assessment of miRNA expression signatures in exosomes is a promising tool for cancer research and clinical diagnosis. In this study, we report the different miRNA signatures and identified several deregulated miRNA in BC patients with ALN metastasis compared with those without ALN metastasis. We identified that the level of exosomal *miR‐363‐5p* in ALN‐positive BC patients was significantly lower than that in ALN‐negative patients.

Evaluation of miRNA expression in tumor tissues is necessary, as the parallel down‐regulation acts as the logical foundation of a tumor‐derived diagnostic marker and is indispensable for mechanism interpretation. We investigated the *miR‐363‐5p* level in BC tissue of both in‐house patients and external datasets. The results were consistent with that of plasma exosomes. Those patients who were diagnosed with LN‐positive BC had a significantly lower level of *miR‐363‐5p*. These results indicated that deregulated exosomal *miR‐363‐5p* level is associated with transcriptional changes in primary tumor tissue. These changes contribute substantially to LNM in BC.

We performed an *in* *silico* diagnostic test and verified that *miR‐363‐5p* alone has an AUC of 0.733–0.958 in predicting LNM in multiple independent datasets. Previous studies have shown that imaging approaches, namely, axillary ultrasound and MRI, perform similarly performance ALN staging; the AUC of MRI alone was 0.665 [33, 34]. We consider that exosomal *miR‐363‐5p* can help elevate the accuracy of clinical prediction models if taken into consideration. Furthermore, survival analysis revealed that patients with lower *miR‐363‐5p* have a significantly worse prognosis, especially in node‐negative patients at their initial diagnosis, suggesting that patient stratification using *miR‐363‐5p* can help distinguish individuals with a high risk of BC death. Node‐negative patients with low *miR‐363‐5p* levels might consider adjuvant endocrine therapy.

In this study, we also investigated the functional significance of *miR‐363‐5p*. We found that restoration of *miR‐363‐5p* using mimics significantly inhibited BC cell migration, while it did not appear to affect proliferation. Studies have revealed that low *miR‐363* expression is associated with carcinogenesis and metastasis [35, 36]. Overexpression of *miR‐106a‐363* cluster (*miR20b*, *miR‐363‐3p* and *miR‐363‐5p*) exhibited an anti‐proliferative effect on cancer cells [37]. This indicated that *miR‐363‐5p* would impact the migration ability instead of the proliferation. In combination with the data of this study, we hypothesized that miR‐363 with its mature forms could cause opposite effects on cell proliferation and migration. The expression level of miR‐363 may be upregulated during tumorigenesis, which is associated with increased proliferation in early cancer. Whereas miR‐363 was downregulated during metastasis formation along with the phenotype switch from proliferation to migration, the anti‐migration effect of *miR‐363‐5p* is likely transmitted by circulating exosomes secreted by the primary tumor. Our previous study constructed a prognosis model of node‐negative patients, based mainly on receptor status and tumor size [38]. The miRNA signature can provide distinct tumor information on the tumor’s cellular and molecular characteristics and would increase the accuracy of clinical prediction models. Our functional study supported *miR‐363‐5p* as a specific complementary predictor for LNM as well as patient prognosis.

It has been reported that *miR‐363‐5p* modulates endothelial cell‐specific genes, including angiocrine factors [39], which is consistent with our results. We found that *miR‐363‐5p* regulates *PDGFB* by binding to its 3′‐UTR, which inhibits the activation of PDGF/PDGFR‐related pathways. It is reported that the metastatic potential of mammary epithelial cells depends on the PDGF‐PDGFR loop [40]. PDGF autocrine activates *STAT1* and other pathways, contributing to the induction and maintenance of the EMT in BC. *PDGFB* and dimer protein PDGF‐BB is an important lymphangiogenic factor and contributes to cancer lymphatic metastasis by stimulating MAP kinase activity [41, 42]. *PDGFB* exhibited both proliferative and chemotactic effects on lymphatic endothelial cells and directly caused lymphatic metastasis in BC‐bearing mice [43]. In summary, *miR‐363‐5p*/*PDGFB* might play a pivotal role in BC carcinogenesis and progression, especially related to LN staging. Furthermore, *miR‐363‐5p* might represent a relatively downstream element in a complicated regulation network; however, the different pathways involved in this process require further exploration.

Nevertheless, this study has several limitations. First, the present study only included ER^+^ HER2^−^ patients, and further verification is necessary for other molecular types. Secondly, the sample size of our discovery cohort is relatively small. However, the potential significance of the exosomal *miR‐363‐5p* in BC LNM has been shown.

## 5 Conclusion

In conclusion, our study identified exosome miRNA markers that help evaluate LN status in a noninvasive manner. Exosomal *miR‐363‐5p* showed good accuracy and was confirmed with a functional and molecular basis. These results indicate that exosomal *miR‐363‐5p* may be applicable in developing liquid biopsy strategies to diagnose LNM in BC effectively.

## Ethics approval and consent to participate

All participants signed a written informed consent. Ethics approval for the study was obtained from the Research Ethics Committee of CHCAMS (reference number: NCC2017G‐075).

## Conflict of interest

The authors declare no conflict of interests.

## Author contributions

Xin Wang established the study concept and coordinated laboratory assays. TQ, MZ and SB wrote the manuscript with support from Xin Wang, HC and JL. SB and HZ performed bioinformatics analysis. HC supervised laboratory assays. ZX, YL, XM, CW, JW and HG performed and supervised sample collection. JL coordinated the research. MZ and Xiang Wang contributed to the design and implementation of the research. All authors contributed to data interpretation, and read and approved the final manuscript.

## Supporting information

**Fig. S1**. Characterization of exosomes from the plasma of BC patients. (A) TEM displayed a cup‐shaped exosome. Scale bar: 200 nm. (B) Exosome markers confirmed by western blot indicating the presence of TSG101 and CD63 but the absence of calnexin. (C) NTA analysis revealed the main peak of 85.5 nm

**Fig. S2**. Association of *miR‐363‐5p* expression and LN status in different BC subtypes. The *miR‐363‐5p* expressions are significantly lower in nodal positive BC, exclusively in ER^+^ BC samples.

**Fig. S3**. Tissue expression of *miR‐363‐5p* in in‐house BC tissue and matched para‐tumor tissue. The qPCR results indicated that expression levels of *miR‐363‐5p* in BC are significantly higher than in normal tissues. *P < 0.05 determined by Student’s *t*‐test.

## Data Availability

The datasets generated during the current study are not publicly available as the informed consent does not cover open data disclosure. Access to the data is available from the corresponding author on reasonable request according to approval from the ethical and data protection board.
