# Supplementary figures and images for: Circulating exosomal miR‐363‐5p inhibits lymph node metastasis by downregulating PDGFB and serves as a potential noninvasive biomarker for breast cancer
Source: Mol Oncol. 2021 Jun 25;15(9):2466–79. doi: 10.1002/1878-0261.13029 (PMC8410538; doi:10.1002/1878-0261.13029)

A

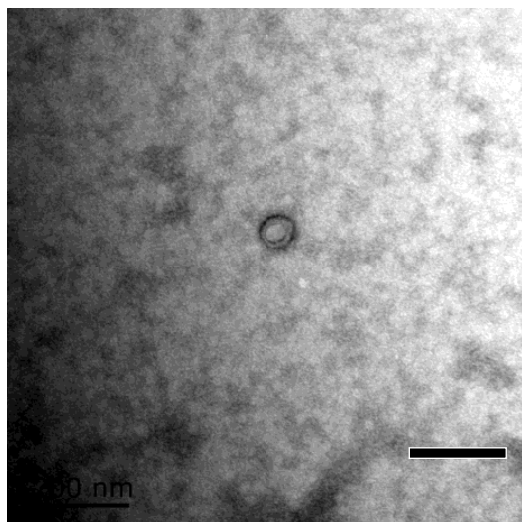

B

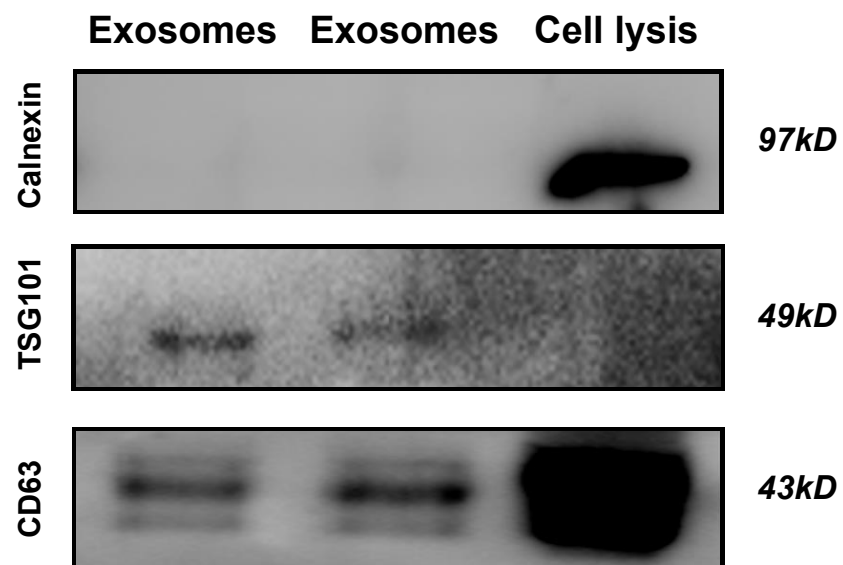

C

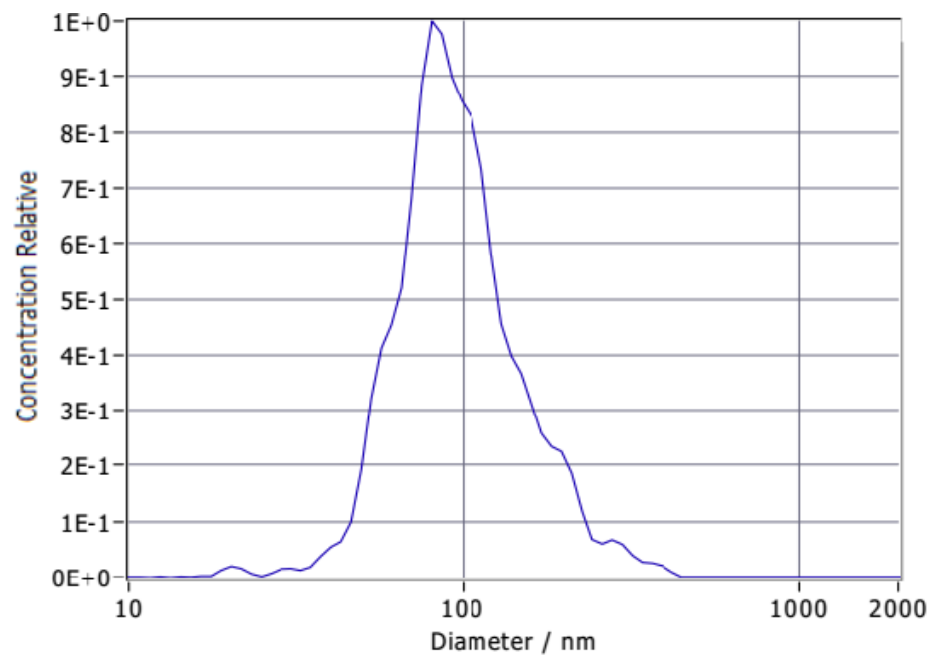

Supplement: Supplementary file 1 — Fig. S1. Characterization of exosomes from the plasma of BC patients. (A) TEM displayed a cup‐shaped exosome. Scale bar: 200 nm. (B) Exosome markers confirmed by western blot indicating the presence of TSG101 and CD63 but the absence of calnexin. (C) NTA analysis revealed the main peak of 85.5 nm [file MOL2-15-2466-s003.pdf]

TCGA hsa-miR-363 TPM

ER-

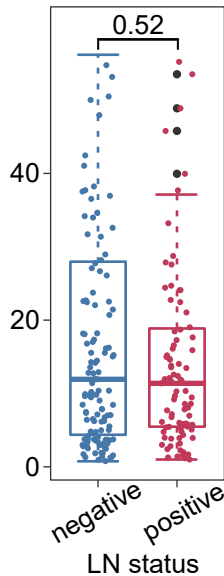

ER+

TCGA hsa-miR-363 TPM

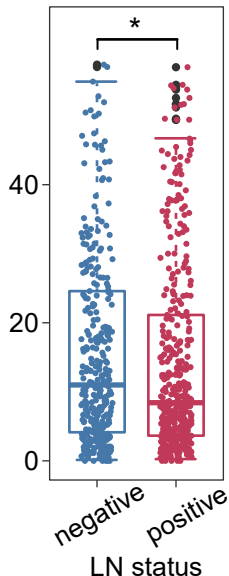

HER2-

TCGA hsa-miR-363 TPM

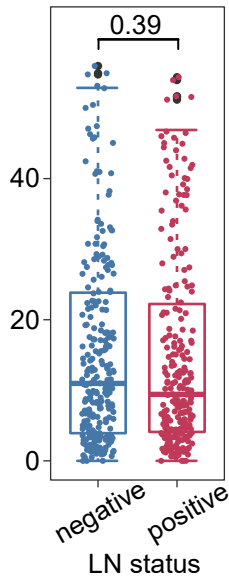

HER2+

TCGA hsa-miR-363 TPM

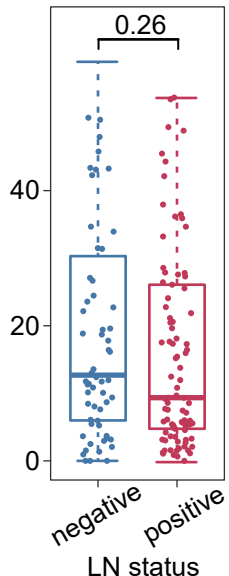

Supplement: Supplementary file 2 — Fig. S2. Association of miR‐363‐5p expression and LN status in different BC subtypes. The miR‐363‐5p expressions are significantly lower in nodal positive BC, exclusively in ER+ BC samples. [file MOL2-15-2466-s002.pdf]

relative miR-363-5p expression

2

1

0.04

Para-tumor

BC

Tissue Type

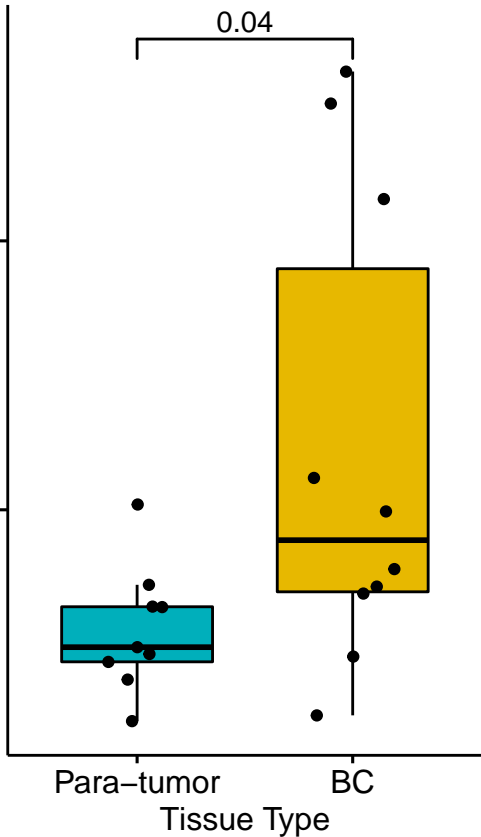

Supplement: Supplementary file 3 — Fig. S3. Tissue expression of miR‐363‐5p in in‐house BC tissue and matched para‐tumor tissue. The qPCR results indicated that expression levels of miR‐363‐5p in BC are significantly higher than in normal tissues. *P < 0.05 determined by Student’s t‐test. [file MOL2-15-2466-s001.pdf]
